# Sperm Chromatin-Induced Ectopic Polar Body Extrusion in Mouse Eggs after ICSI and Delayed Egg Activation

**DOI:** 10.1371/journal.pone.0007171

**Published:** 2009-09-29

**Authors:** Manqi Deng, Rong Li

**Affiliations:** Stowers Institute for Medical Research, Kansas City, Missouri, United States of America; Brunel University, United Kingdom

## Abstract

Meiotic chromosomes in an oocyte are not only a maternal genome carrier but also provide a positional signal to induce cortical polarization and define asymmetric meiotic division of the oocyte, resulting in polar body extrusion and haploidization of the maternal genome. The meiotic chromosomes play dual function in determination of meiosis: 1) organizing a bipolar spindle formation and 2) inducing cortical polarization and assembly of a distinct cortical cytoskeleton structure in the overlying cortex for polar body extrusion. At fertilization, a sperm brings exogenous paternal chromatin into the egg, which induces ectopic cortical polarization at the sperm entry site and leads to a cone formation, known as fertilization cone. Here we show that the sperm chromatin-induced fertilization cone formation is an abortive polar body extrusion due to lack of spindle induction by the sperm chromatin during fertilization. If experimentally manipulating the fertilization process to allow sperm chromatin to induce both cortical polarization and spindle formation, the fertilization cone can be converted into polar body extrusion. This suggests that sperm chromatin is also able to induce polar body extrusion, like its maternal counterpart. The usually observed cone formation instead of ectopic polar body extrusion induced by sperm chromatin during fertilization is due to special sperm chromatin compaction which restrains it from rapid spindle induction and therefore provides a protective mechanism to prevent a possible paternal genome loss during ectopic polar body extrusion.

## Introduction

Sexual reproduction is the formation of a new individual following the union of two haploid gametes, one from a male and the other from a female, through a process of fertilization. It is known that male and female germ cells undergo different processes of meiotic divisions and post-meiotic remodeling to produce gametes with striking differences in size, shape, chromatin packaging state, cell cycle, and etc. An oocyte undergoes two rounds of extreme asymmetric meiotic division following one round of DNA replication, producing a large sized haploid egg and discarding the other half of the chromosomes into two small polar bodies designated for degeneration. Female meiosis in many animal specieses is not complete at ovulation but is arrested at metaphase of the second meiosis (MII), waiting for fertilization to reinitiate the meiosis II. At fertilization, a sperm not only delivers a haploid paternal genome to the egg but also triggers resumption and completion of meiosis II by inducing Ca^2+^ spikes [1], culminating in the extrusion of the second polar body (PbII). In contrast to female meiosis, a spermatocyte produces 4 haploid spermatids after completion of meiosis. However, successful haploidization of spermatocytes is only the first step towards gamete production and the produced haploid spermatids must undergo extensive post-meiotic chromatin reorganization and morphological changes before fertilization. One of the most striking post-meiotic changes is to repackage the paternal haploid genome with the testis-specific nuclear basic proteins, e.g. protamines, forming a tightly compacted chromatin structure [2], [3], [4], [5], [6] coupled with dramatic morphological changes from round spermatids into tadpole-shaped spermatozoa with motility [7]. The protamine-packed DNA makes the sperm chromatin six-fold more compacted than the somatic histone-chromatin [3], [8] and the compaction is further enhanced by crosslinking (via disulfide bonds) while passing through epididymis before ejaculation [9], [10].

Because of the special DNA packaging and testis-specific chromatin remodeling during spermatogenesis, sperm chromatin behaves differently from its female counterpart during fertilization and early embryo development [7], [11], [12], [13], [14], [15], [16], [17], [18]. The unique post-meiotic sperm chromatin remodeling coupled with morphological changes have been thought to be important for packaging the paternal haploid genome into a highly compacted nucleus which is critical for the maintenance of chromatin integrity, sperm motility and fertilization [7], [18], [19], [20], [21], [22]. Indeed, genetic disruption of the post-meiotic chromatin remodeling processes often compromises sperm motility and fertilization, which leads to infertility. Because of this, it is difficult to evaluate the biological functions of the special post-meiotic sperm chromatin reorganization during fertilization as well as the fertilization-triggered PbII extrusion in the eggs. By taking advantage of the techniques of intracytoplasmic sperm injection (ICSI) and artificial activation of eggs, we can study the question by separating sperm-egg fusion with egg activation and ask what if a sperm delivers a somatic-like chromatin, instead of the protamine-packaged chromatin to the egg at fertilization. It is known that injection of immature sperm at round spermatid stage (which is unable to induce Ca^2+^ oscillations required for egg activation) [23] into an egg and artificial activation of the egg can result in successful fertilization and post-fertilization development to term albeit at a low rate [23], [24]. This supports the notion that most if not all of the biochemical and morphological changes in the sperm may be to help the sperm to acquire the ability to penetrate and activate the eggs that can be bypassed by ICSI and artificial egg activation. Probably the other significant role of paternal genome remodeling is imprinting, which is critical for normal embryo development to term [5], [25]. However, it seems that the paternal gene imprinting occurs early during spermatogenesis, well before the post-meiotic chromatin reorganization, since injection of round spermatids or even earlier stages of primary spermatocyte into mouse eggs can participate in fertilization which supports the post-fertilization development to term albeit with low percentages [26], [27]. All these suggest that the special male-specific sperm chromatin reorganization may have other currently unidentified functions during fertilization. In this study we used ICSI and artificial egg activation to investigate if the special post-meiotic remodeling of sperm chromatin plays any additional roles during the process of fertilization.

We and others showed previously that the cortex of mouse eggs is inductive to a general chromatin signal by inducing assembly of a distinct cortical structure, characterized by formation of a cortical actin cap [28], [29] enclosed by a myosin II ring (Deng et al., 2007). Formation of this structure is coupled with reorganization of cortical granules, surface microvilli, PAR proteins on the overlying cortex to form a cortical domain free of cortical granules and microvilli, rich in actomyosin and also PAR proteins in mouse oocytes [29], [30], [31], [32], [33]. Like the maternal chromosomes, sperm chromatin induces formation of a similar cortical structure during fertilization [28], [34], [35], [36], [37] or ICSI [36], [37]. It was observed long time ago that the sperm-induced cortical structure protrudes and forms a cone during fertilization, called fertilization cone [28], [34], [35], [38]. It was initially thought that the fertilization cone may facilitate sperm incorporation into the fertilizing eggs. However, block of fertilization cone formation by depolymerizing actin filaments has little effect on sperm incorporation into the mammalian eggs during IVF [28], [39], [40], [41]. For many years, we are puzzled by the sperm-induced cortical cone formation during fertilization, with little knowledge on the mechanism, function and its relevance to polar body extrusion. Our new results suggest that the sperm-induced cone formation may just be a sign of incomplete polar body extrusion due to lack of spindle induction by the fertilizing sperm and hence spindle midzone signals for completion of cytokinesis [42], [43]. The sperm-induced cone can be converted into complete polar body extrusion if egg activation is delayed to allow sperm chromatin to induce a spindle in the egg. These findings may reveal a previously unappreciated mechanism to prevent the sperm chromatin-induced ectopic polar body extrusion during fertilization. The results may provide new thoughts on understanding the mechanism of fertilization and particularly ICSI when using round spermatids or earlier stage spermatocytes to treat human infertility.

## Results

### Sperm chromatin induced polar body-like cortical cone formation in the eggs during fertilization

**Figure 1:**
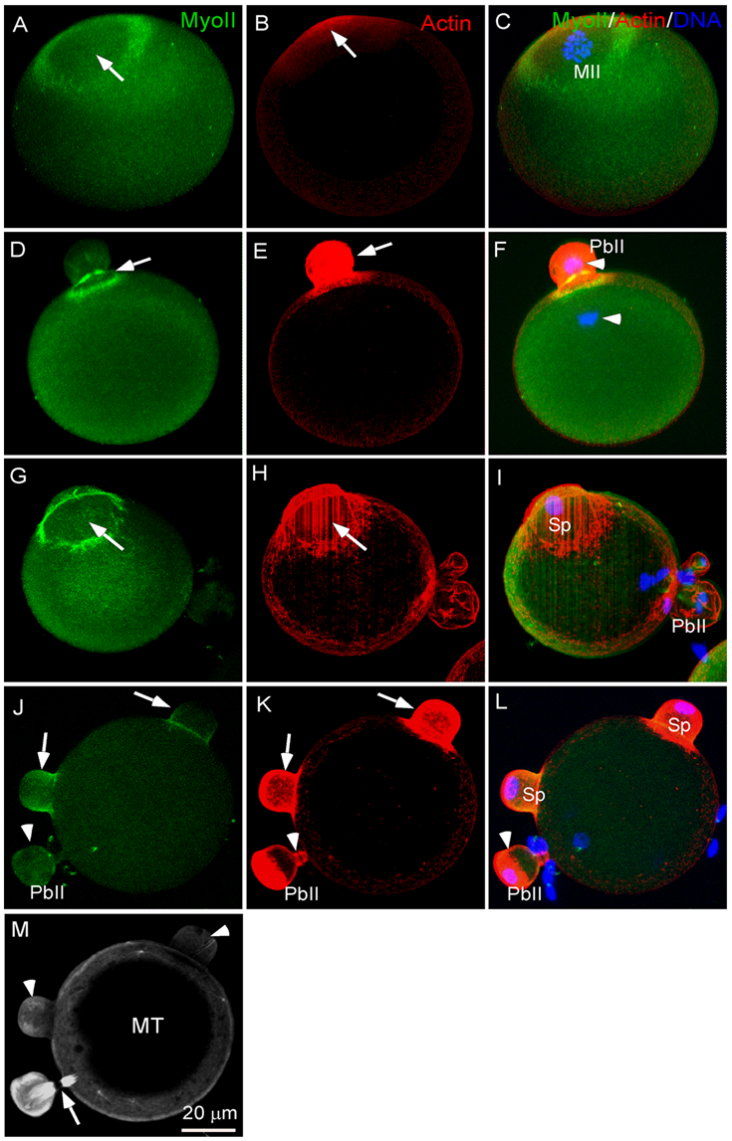
Comparison of the maternal chromosome-induced polar body extrusion and the sperm chromatin-induced cortical cone formation during fertilization. (A–C) MII chromosome-induced cortical assembly of a MyoII ring (A, arrow) and an actin cap (B, arrow) in the overlying cortex (C, merged image). The shown images are representatives from observation of over 50 MII eggs. (D–F) Cortical actomyosin reorganization during second polar body extrusion (PbII). Note the pinching-off of PbII (arrows) and the segregated meiotic chromosomes (arrowheads in F). Eggs were activated by SrCl_2_. The shown images are representatives from observation of 21 eggs. (G–I) During fertilization, sperm chromatin (Sp) induces a similar cortical actomyosin structure (arrows) which protrudes and forms the fertilization cone. The extra DAPI-stained structures close to PbII are surface-bound sperm. The shown are representative images from observation of 32 eggs. (J–L) Two fertilization cones (arrows) induced by two penetrated sperm (Sp). Note the similarity between PbII, (arrowhead) and the sperm chromatin-induced fertilization cones (arrows), except the cytoplasmic abscission observed only in PbII (K, arrowhead). (M) Microtubule (MT) staining showing the same egg in J–L. Note that the sperm chromatin did not induce spindle formation (arrowheads). The arrow indicates the maternal spindle midzone. The shown images are representatives from observation of 11 dispermy eggs. In all the images, MyoII is shown in green, actin in red and DNA in blue except in M where MT is shown in white. All the experiments were repeated at least three times unless otherwise specified in the text.

**Figure 2:**
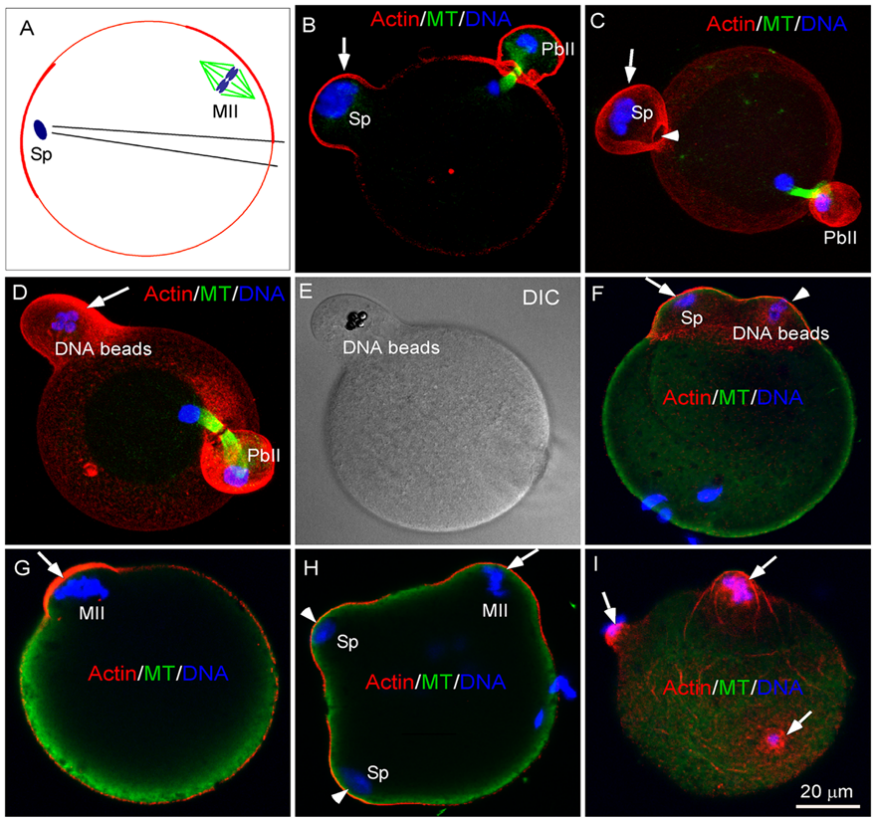
Induction of cone formation by chromatin from various origins. (A) A diagram depicting microinjection of sperm chromatin or DNA beads into a subcortex of an egg distant from the MII spindle. (B, C) Injection of sperm chromatin and subsequent egg activation by SrCl_2_ induced cortical cone formation (arrows) similar to that induced by IVF. Note that without induction of spindle formation, the sperm chromatin-induced cortical cone failed to complete cytoplasmic abscission, (C, arrowhead). The ICSI experiments were repeated three times and the shown images are representatives from observation of 35 eggs. (D, E) DNA bead-induced cone formation. D and E show a confocal and a DIC image of the same egg respectively. The DNA bead injection experiments were repeated four times and the shown images are representatives from observation of 17 eggs. (F) An egg injected with DNA beads was activated by IVF. Note that DNA beads (arrowhead) and the penetrated sperm (arrow) induced two similar cones in the same egg (Shown is a representative image of 21 analyzed eggs). (G–I) MII chromosomes, sperm chromatin and scattered chromosomes induced similar cone formation in the presence of 1 µM nocodazole. (G) An MII chromosome-induced cone (Shown is a representative image from observation of 42 treated eggs). (H) Sperm chromatin-induced 2 fertilization cones (arrowheads) and an MII chromosome-induced cone in the same egg during IVF (observation of 23 fertilized eggs treated with nocodazole). (I) Three cones (arrows) induced by the scattered MII chromosomes after nocodazole treatment in a SrCl_2_-activated egg (observation of 37 treated eggs).

Chromosomes play important roles in determining asymmetric meiotic divisions in the oocytes by inducing spindle formation and establishing a special cortical domain, characterized by formation of an actin cap surrounded by a myosin ring and exclusion of cortical granules and microvilli from the region (Figure 1A–C, staining of microvilli is not shown) [28], [30], [33], [34], [38]. This actomyosin-based cortical structure is instrumental for polar body extrusion (Figure 1D–F) and disruption of it blocks polar body extrusion (data not shown) [28], [35], [44]. During fertilization, the sperm chromatin induced a similar cortical structure (Figure 1G–I, arrows) as that induced by the maternal MII chromosomes (Figure 1A–C). It is interesting to note that while the maternal chromosome-induced cortical cap underwent polar body extrusion (Figure 1J–L, arrowheads) the paternal chromatin-induced cortical cap underwent just cortical protrusion, forming a polar body-like structure (Figure 1J–L, arrows), called fertilization cone [28], [34], [35], [38]. Unlike polar body extrusion induced by the meiotic chromosome/spindle, the sperm chromatin-induced cortical cones never progressed to the point of cytoplasmic abscission (Figure 1K, arrows vs. arrowhead, Figure 2B, C) and eventually regressed by the time of pronuclear formation (data not shown). The failure of the sperm-induced fertilization cones to complete cytokinesis might be due to the fact that no spindle was induced by the sperm chromatin (Figure 1M, arrowheads vs arrow) [30] as spindle midzone signals are required for cytokinetic abscission [42], [45]. These results raise some interesting questions and prompt us to make some assumptions. Is there a mechanistic link between fertilization cone formation and polar body extrusion? Why maternal and paternal chromatin induce the similar cortical structures which have different developmental fates and what is the function of sperm-induced cone formation? Is the observed sperm-induced cortical cone specific to sperm-egg interaction or a general chromatin-induced cortical response? Can the sperm chromatin induced-cortical cone be converted to ectopic polar body extrusion?

### Fertilization cone formation is a general chromatin-induced cortical response in the activated eggs

To determine if the observed fertilization cone formation was due to sperm-egg membrane fusion during fertilization, we injected demembraned sperm chromatin into egg cortex distant from the pipette penetration site (depicted in Figure 2A) and immediately activated the eggs with SrCl_2_ which mimics the fertilization-induced Ca^2+^ oscillations to induce egg activation [46], [47], [48]. The injected sperm chromatin induced similar cone formation in the overlying cortex (Figure 2B, C, arrows) but not the pipette penetration site from the opposite cortex, suggesting that the observed cone formation is unlikely due to membrane disturbance by microinjection from the opposite cortex. To test if cortical cone formation is specifically induced by sperm chromatin, we injected beads coated with plasmid DNA [33], [49] into eggs and activated the eggs as described above. The injected DNA beads which can assemble chromatin structures in the egg cytoplasm [49] were fully capable of inducing actin cap formation as we showed previously [33]. Strikingly, the injected DNA beads induced comparable cortical cone formation as that induced by the sperm chromatin (Figure 2D–F) in the absence of spindle formation, suggesting that cone formation is a general chromatin-induced cortical response during egg activation. Injection of non-DNA coated beads was unable to induce either cortical cap or cone formation (data not shown, [33]). Disruption of actomyosin assembly or its contractility abolished both sperm chromatin-induced cone formation and polar body extrusion (data not shown) [28], [35], [44], suggesting that the two events are driven by contraction of the cortical actomyosin structures that are induced by chromatin. To determine if the presence of spindle determines the fate of chromatin-induced cortical caps, either forming a fertilization cone or a polar body, we used nocodazole (1 µM) to depolymerize the maternal spindle microtubules, which is known to have no effect on the chromatin-induced cortical cap [30], [33]. It is interesting to see that after disruption of the MII spindles the maternal chromosome-induced caps failed to complete polar body abscission but instead, formed cortical cones (Figure 2G, 2H, arrows) which are comparable to the sperm-induced fertilization cones (Figure 2H, arrowheads), suggesting that the abscission of polar body requires spindle signals. In some eggs, nocodazole resulted in maternal chromosome scattering to the egg cortex [30], [50], inducing multiple cone formation (Figure 2I, arrows) which are also comparable to the sperm-induced fertilization cones. Taken together, these results suggest that cone formation is a general chromatin-induced cortical response during egg activation. The sperm chromatin-induced cone formation may represent an abortive polar body extrusion due to lack of spindle midzone signals which are known to be required for the completion of cytokinesis during mitosis [51], [52], [53]. Nevertheless, there has been no evidence showing that the sperm chromatin-induced cortical cones can be converted to polar body extrusion if experimentally installing a bipolar spindle under beneath the cone.

### Sperm chromatin is capable of inducing both cortical polarization and bipolar spindle formation and leads to ectopic polar body extrusion

**Figure 3:**
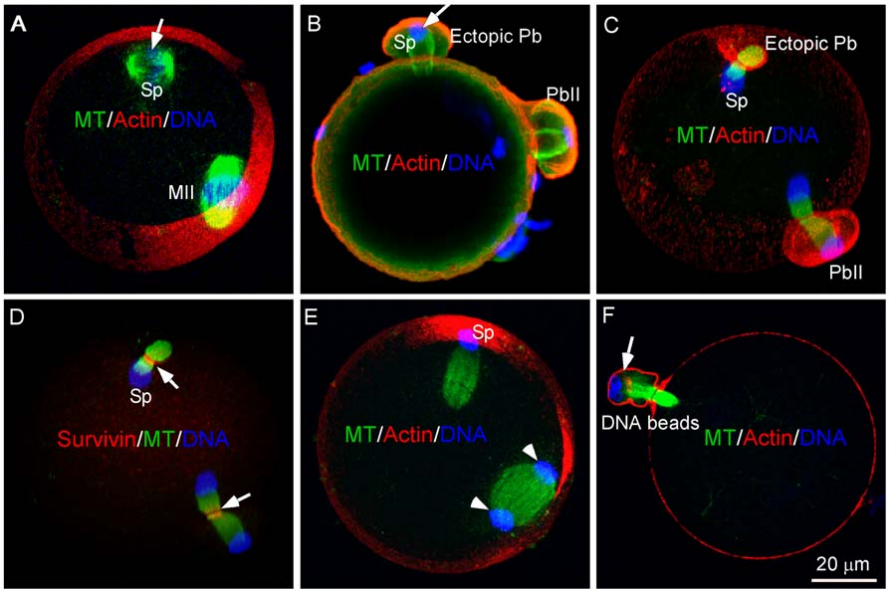
Induction of ectopic polar body extrusion by sperm chromatin and DNA beads. (A) Sperm chromatin induced both cortical actin cap and bipolar spindle formation after injected into an MII egg for 3–4 h (Shown is a representative image from observation of over 100 sperm chromatin-injected eggs). (B) Sperm chromatin/spindle-induced ectopic polar body extrusion (arrow) compared with MII spindle-induced PbII extrusion after fertilization. The fertilizing sperm that activated the egg is out of focus and not shown in the image (observation of 33 IVF eggs). (C) Sperm chromatin/spindle-induced ectopic polar body extrusion in a SrCl_2_-activated egg (observation of 55 eggs). (D) Both MII spindle and sperm chromatin-induced spindle underwent typical morphological changes after anaphase onset and formed distinct spindle midzones revealed by a midzone marker, survivin (red, arrows) (a representative image from observation of 51 eggs). (E) Mono-polar movement of sperm chromatin mass during ectopic polar body extrusion compared with equal segregation of the MII chromosomes (arrowheads) (observation of 43 eggs). (F) DNA bead-induced ectopic polar body extrusion (arrow) in a SrCl_2_-activated egg (a representative image from observation of 63 eggs).

Earlier research has shown that mouse sperm chromatin is able to induce bipolar spindle formation after exposing to metaphase cytoplasm for enough time [54], [55], [56], which is in contrast to the observation of only a half spindle formation induced by a single *Xenopus* sperm chromatin in egg extracts [57], [58]. This suggests that the mouse haploid sperm chromatin is fully capable of inducing bipolar spindle formation in oocytes in the absence of centrosomes. The lack of spindle induction by sperm chromatin during normal fertilization may be due to the fact that spindle induction by sperm chromatin requires a longer time (3–4 h) than that for cortical cap induction (1 h) [33]. To test this possibility further, we injected sperm chromatin into MII eggs and cultured the eggs for 2–3 h to allow spindle formation around the sperm chromatin (Figure 3A). Subsequent activation of these eggs by SrCl_2_ resulted in synchronous extrusions of two polar bodies, one induced by the MII chromosomes and the other induced by the sperm chromatin (Figure 3B, C). The sperm-spindle underwent similar and synchronous metaphase-anaphase transition as the MII spindle and formed a distinct anaphase spindle midzone (Figure 3D, arrows).The ectopic polar body induced by the sperm chromatin had completed cytoplasmic abscission (supplemental data Figure S1). It is interesting to note that the whole sperm chromatin mass moved together to one spindle pole (Figure 3B–E) and in most cases, expelled into the ectopic polar body (Figure 3B). The observed non-disjunction of sperm chromatin during ectopic polar body extrusion makes it easy to distinguish the maternal chromosome-induced PbII from the sperm chromatin-induced ectopic polar body. Actually, if allowing DNA beads to induce both cortical cap and spindle formation prior to egg activation, they also led to ectopic polar body extrusion (Figure 3F, arrow, [59]), suggesting that induction of polar body extrusion is not unique to the maternal meiotic chromosomes and any chromatin structures can induce polar body extrusion in the eggs.

### De novo induction of spindle and cortical cap formation by chromosomes after egg activation

**Figure 4:**
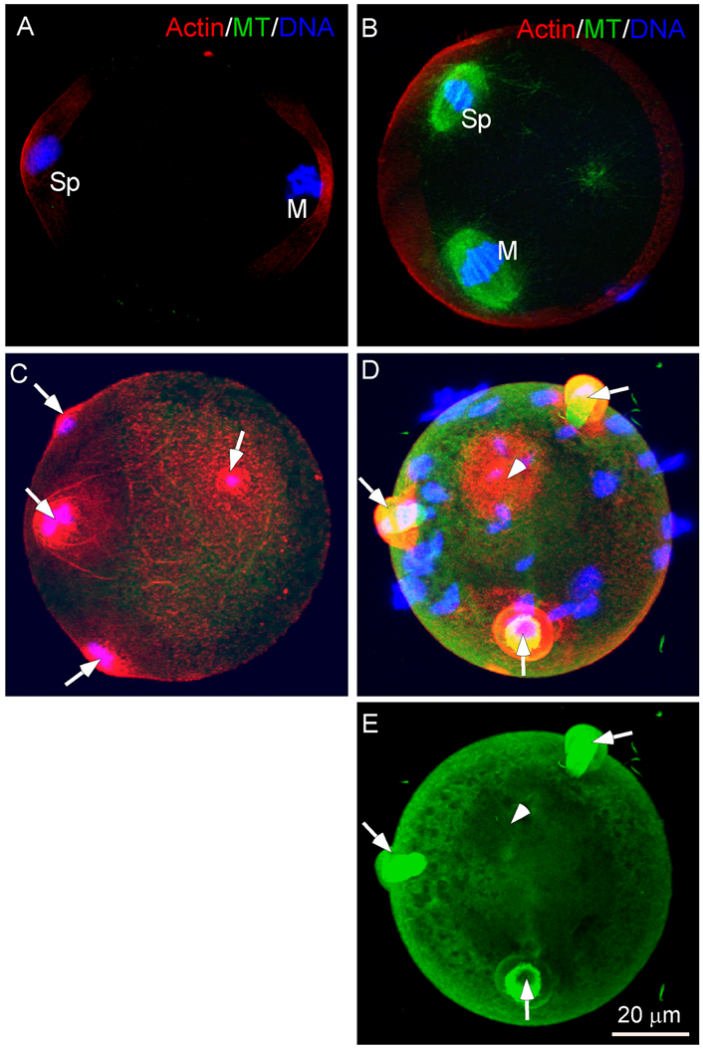
De novo spindle formation induced by chromatin after egg activation. (A, B) The sperm-injected eggs were treated with nocodazole to disassemble all the pre-formed spindles prior to egg activation treatment. Nocodazole was then washed out and eggs were activated by SrCl_2_. The images were taken at 0 min (A, a representative image from observation of 54 eggs) and 30 min (B, a representative image from observation of 44 eggs) time points after washing out of nocodazole and subsequent SrCl_2_ treatment. Note that two spindles were re-assembled, each around the maternal (M) and the sperm (Sp) chromatin at the 30 min time point. (C, D) Nocodazole-induced maternal chromosome scattering, which induced multiple cortical caps and cones in a SrCl_2_-activated egg (C, arrows, a representative image from observation of 37 eggs) which underwent multiple polar body extrusions after washing out nocodazole and subsequently activated with SrCl_2_ for 2–3 h (D, arrows, a representative image from observation of 18 eggs). (E) MT image of D. Note that the scattered maternal chromosomes induced three polar bodies, each with a defined spindle (D, arrows), and one cortical cone with no spindle formation underlying (arrowhead).

The above experiments suggest that introduction of exogenous chromatin into MII eggs can induce both cortical cap and spindle which can result in ectopic polar body extrusion after egg activation. To make this relevant to the situation of fertilization during which a sperm chromatin is introduced into the egg and a series of Ca^2+^ oscillations are also induced almost simultaneously [60], it has to be shown that eggs can still support both cortical cap and spindle formation after initiation of egg activation. To test if eggs can still support de novo spindle formation after inducing Ca^2+^ oscillations, we first used nocodazole to disassemble the pre-formed spindles that were induced by either maternal chromosomes or possibly by sperm chromatin after injected into eggs for 2 h. We then washed out the drug and activated the eggs immediately with SrCl_2_ or by in vitro fertilization. Spindles were observed to re-assemble around both the maternal chromosomes and the sperm chromatin in 30 min (Figure 4A, B). In some eggs, nocodazole-induced disassembly of the meiotic spindle caused scattering of maternal chromosomes which induced formation of multiple cortical caps (Figure 4C, arrows) and spindles surrounding each chromosome fragment after washing out the drug (Figure 4D, E, arrows), which resulted in multiple polar body extrusions (Figure 4D, arrows). These results are consistent with the previous observation that IVF of eggs in nocodazole for 2 h and subsequent washing out of nocodazole results in multiple polar body extrusion [30]. This suggests that the chromatin-induced cortical cap and spindle formation can be faster than the Ca^2+^-induced cell cycle progression form metaphase to anaphase. Taken together, these results justify for a concern that ectopic polar body extrusion could be induced by sperm chromatin during fertilization especially by ICSI.

### Different sperm chromatin organization and compaction affects the speed of spindle induction in the eggs

**Figure 5:**
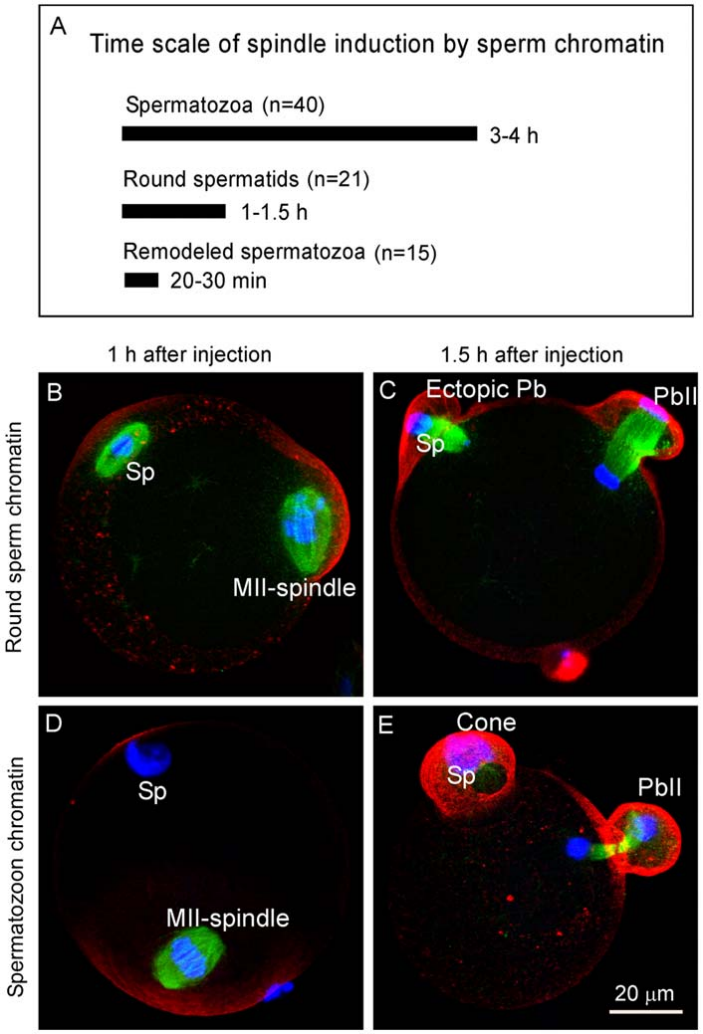
Accelerated spindle formation induced by injection of less compacted sperm chromatin and ectopic polar body extrusion in the eggs. (A) A comparison of time required for spindle induction by injection of sperm chromatin at different organization states. (B, C) Representative images showing the time course of spindle formation induced by injection of round spermatid chromatin into the SrCl_2_-activated eggs. Note that the round spermatid chromatin induced a bipolar spindle 1 h after injection which resulted in ectopic polar body extrusion (n = 17/35). (D, E) Control experiment by injection of regular spermatozoon chromatin which failed to induce spindle formation at the same time point (D, observation of over 100 eggs) and only induced cone formation at the 1.5 h time point (E, observation of 65 eggs).

The differences in the speed of spindle induction by maternal and paternal chromatin may reflect the fact that the chromatin prepared from mature spermatozoa is highly compacted by protamines during spermatogenesis [2], [3], [7] and requires extensive decondensation and re-condensation before acquiring the ability to induce spindle formation [54], [55], [56]. To test if somatic histone-packaged sperm chromatin induces faster spindle formation than the protamine-packaged sperm chromatin, demembraned round spermatids which have not yet started the post-meiotic chromatin remodeling [7], were injected into MII eggs. Indeed, they induced faster spindle formation than the chromatin prepared from spermatozoa (Figure 5A, B vs. Figure 5D). This is consistent with the time frame to transform the injected round spermatids nuclei into the metaphase chromosomes in MII eggs [23], [26]. Injection of somatic nuclei derived from cumulus cells at G1 stage into eggs also led to faster spindle formation than that induced by spermatozoon chromatin [56] (data not shown). If the chromatin of spermatozoa was first remodeled in MII eggs for 2–3 h and then injected into another egg, it could induce spindle formation in 20–30 min (Figure 5A). This suggests that different chromatin organization (by either conventional histones or protamines) and compaction state affect the speed of spindle induction in the eggs.

Accordingly, injection of round sperm and subsequent egg activation resulted in ectopic polar body extrusion in 17 out of 35 injected eggs (Figure 5B, C). In the control experiment, injection of mature spermatozoon chromatin into eggs and subsequent egg activation failed to induce spindle formation or ectopic polar body extrusion at the same time points (Figure 5D, E, observation of over 100 eggs) but only induced cone formation (Figure 5E). This suggests that the special repackaging of sperm chromatin with protamines is critical for delaying spindle formation and preventing possible paternal chromosome loss during ectopic polar body extrusion.

## Discussion

Sperm-induced fertilization cone has been observed for decades but little is known about the mechanism of formation and particularly its relevance to polar body extrusion. Our results suggest that the sperm chromatin-induced fertilization cone and meiotic chromosome-induced polar body extrusion may share the same cytokinetic mechanism. The sperm chromatin-induced cortical cone can result in ectopic polar body extrusion if sperm chromatin is allowed to assemble a bipolar spindle prior to anaphase onset. Conversely, the meiotic chromosome-induced polar body extrusion can be directed to cone formation if the meiotic spindle is not formed or disrupted. This suggests that the chromatin-induced either cortical cone formation or polar body extrusion can be viewed as at different stages of cytokinesis. During polar body extrusion, cortical cone formation is spatially coordinated with anaphase spindle midzone-induced membrane furrowing and abscission which drives cytokinesis to completion. While in the absence of spindle, the cortical cone can not be abscissed in the absence of midzone signals.

The entire egg cortex possess a remarkable ability to assembly the distinct actomyosin structure in response to a nearby chromatin signal (even DNA-coated beads), enabling a robust extrusion of unwanted maternal DNA during meiosis. However, induction of ectopic polar body by a fertilizing sperm chromatin poses a risk of paternal genome loss, as we have shown that sperm chromatin has a high frequency of being extruded with the ectopic polar body. This would generate a situation reminiscent of gynogenesis, where the paternal genome is eliminated from the egg after fertilization [61]. Even partial sperm chromatin loss during fertilization would be detrimental because embryos with aneuploidy frequently lead to miscarriages or birth defects. Our results suggest that the key mechanism that prevents the paternal chromosome loss during fertilization may be to specially package the paternal chromatin in a highly compacted state during spermatogenesis, which prevents the rapid spindle induction by the sperm chromatin in the eggs.

It is well known that sperm chromatin undergoes extensive chromatin remodeling and reorganization after completion of meiosis during spermatogenesis. The paternal genome is repacked with a male-specific nuclear protein, protamine, which makes sperm chromatin highly compacted compared to its maternal counterpart. It has been thought that repackaging of sperm chromatin with protamines is important for preserving DNA integrity [19], [62], [63] during sperm transport in both male and female reproductive tracts to the fertilization site. There are numerous reports showing that defective DNA packing makes sperm chromatin more vulnerable to damage and leads to infertility [19], [62], [63]. However, due to the challenges that disruption of post-meiotic repacking of sperm chromatin during spermatogenesis inevitably leads to loss of sperm motility and therefore fertilization ability, it remains unclear with regard to the specific role of sperm chromatin remodeling during fertilization. Our findings may provide a new clue to understanding the role of special sperm chromatin packaging in fertilization and polar body extrusion, which may have relevance to ICSI used in treating human infertility.

ICSI has been used in treating human infertility for almost twenty years. It should be noted that in most cases, only mature spermatozoa are used for ICSI. Injection of immature round spermatids or earlier stages of spermatocytes into eggs results in very low rates of fertilization and development to term [64], which limits the use of ICSI to cure patients who are incapable of generating mature spermatozoa. As we show in the mouse, injection of chromatin prepared from round spermatids can induce both cortical cap and bipolar spindle in a short time which could result in ectopic polar body and sperm chromatin loss during polar body extrusion. Whereas, injection of well compacted chromatin prepared from spermatozoa only induces cortical cap but not spindle formation during the normal cell cycle window after egg activation. Due to this difference, injection of mature spermatozoa can only allow cortical cone formation while injection of the loosely compacted chromatin from round spermatids and subsequent egg activation increases the chance for ectopic polar body extrusion and possible aneuploidy formation. Thus, proper sperm chromatin post-meiotic packaging may play a previously unappreciated role in preventing ectopic polar body extrusion and aneuploidy formation during meiosis II. It should be pointed out however, that our data are currently limited to the mouse model and it remains to be tested if ICSI with round spermatids would result in higher rates of ectopic polar body extrusion and paternal chromatin loss during polar body extrusion in the humans.

It is interesting to note that while the sperm chromatin-induced spindle formation is discriminatively repressed in the fertilized eggs, induction of cortical polarization by sperm chromatin is not affected. This suggests that the signals that emanate from the chromosomes to induce spindle formation and cortical reorganization are qualitatively and quantitatively different. Regardless, it remains unclear what biological function of the sperm chromatin-induced cortical cone is during fertilization. It appears that sperm incorporation into an egg does not require pre-formation of a cortical cone, and disruption of cone formation does not affect fertilization [28], [39], [41]. It is possible, however, that by inducing assembly of a cortical actomyosin cap/ring structure, and penetrated sperm is restrained to the cortex, and therefore kept away from the MII chromosomes which are poised to undergo meiotic division. Another possible function for restraining sperm chromatin to the egg cortex is that many histones and DNA remodeling factors, such as nucleoplasmin and the DNA methyltransferase Dnmt1, nucleoplasmin-2, etc., are all enriched in the cortex of MII eggs [65], [66] (our observation), which may facilitate efficient epigenetic reprogramming of the differently organized paternal genome for normal embryonic development.

In summary, post-meiotic remodeling and repackaging of sperm chromatin may play a previously unappreciated role in orchestrating cytoskeletal assembly but limiting its ability to induce spindle formation during fertilization, thus constituting a protective mechanism to prevent ectopic polar body extrusion and a potential of paternal genome loss during fertilization.

## Materials and Methods

The experimental animals were handled in accordance with good animal practice as defined by the National Institute of Health of the United States and guidelines of Institutional Animal Care and Use Committee (IACUC) and all the animal work was approved by the IACUC committee at the Stowers Institute for Medical Research, protocol #2007-0013.

### Egg collection and in vitro fertilization

Female mice of CD1 at ages of 4–6 week-old received superovulation treatment by injection of pregnant mare serum gonadotropin (PMSG) and human chorionic gonadotropin (hCG) as described [67]. The ovulated eggs were collected from oviducts at 14–15 h post hCG injection as previously described [36].

For in vitro fertilization, eggs were removed of zona pellucidia by brief treatment in acidic Tyrode solution [67] and inseminated with sperm that were collected from cauda epididymes of male mice and capacitated in Whitten's medium for 1.5 h [67]. After insemination for 2 h, eggs were washed out of surface bound sperm and fixed for immunofluorescence study.

### Preparation of sperm chromatin, DNA beads and round spermatids for microinjection

Mature spermatozoa were collected from cauda epididymes as described above for the in vitro fertilization. The collected sperm were sonicated in Triton X-100 to remove tail and plasma membranes, plus ionic detergent treatment to produce demembraned sperm heads for microinjection [13], [36], [68]. The prepared sperm heads were further heat inactivated to abolish their ability to induce Ca^2+^ oscillations and egg activation [36]. Round spermatids were collected from testis and individual nucleus was isolated by passing through a micropipette to remove the plasma membranes and most of the cytoplasm as described before [23]. Somatic nuclei were prepared from cumulus cells as used for nuclear transfer [48], [69]. DNA beads were prepared as described before [33], [49].

### Microinjection

Microinjection was performed by using micromanipulators (Narishige, Japan) and a Piezol system (PrimeTech, Japan). A single demembraned sperm head, round spermatid nucleus, or a cluster of 3–5 DNA beads were separately injected into the cortex distant from the MII chromosome/spindle, aiming to induce ectopic formation of a cortical cap and a bipolar spindle as described previously [33], [36] for convenient observation of ectopic polar body extrusion or cortical cone formation.

The injected eggs were cultured in M16 (Chemicon) at 37°C in an atmosphere of 5% CO_2_ in air for different periods of time before egg activation to induce anaphase onset and polar body extrusion.

### Egg activation

Eggs were either parthenogenetically activated by using 10 mM SrCl_2_ in Ca^2+^ free CZB [48] or IVF as described above. For convenient observation of chromatin-induced cortical cone formation and polar body extrusion, eggs were removed of zona pellucidia using acidic Tyrode solution [67].

### Immunofluorescence and confocal microscopy

Eggs were fixed, immunostained and mounted on the slides as described previously [33]. All the images were acquired by using a 40X or 63X oil objective on a Zeiss LSM510 confocal microscope. To construct 3D images, a stack of at least 50 Z-section images spanning all the observed structures was collected and reconstructed using Zeiss LSM-CFS. The figures were assembled by importing the images to Photoshop 7.0.

## Supporting Information

Figure S1 Complete cytoplasmic abscission of ectopic polar body induced bt sperm chromatin/spindle. Confocal images showing complete cytoplasmic abscission of the ectopic polar body induced by sperm chromatin-spindle. (A–C) Different confocal sections of an egg showing ectopic polar body at 7 o'clock position (arrows) and PbII at the 11 o'clock position. Note the complete separation of cytoplasm membrane between ectopic polar body (C arrow) and the fertilized egg. The sperm chromatin-injected egg was activated by IVF and the extra DAPI staining spots are surface bound sperm.
(1.10 MB DOC)
