# Supplementary material for: Sperm Chromatin-Induced Ectopic Polar Body Extrusion in Mouse Eggs after ICSI and Delayed Egg Activation
Source: PLoS One. 2009 Sep 29;4(9):e7171. doi: 10.1371/journal.pone.0007171 (PMC2746308; doi:10.1371/journal.pone.0007171)
Supplement: Figure S1 — Complete cytoplasmic abscission of ectopic polar body induced bt sperm chromatin/spindle. Confocal images showing complete cytoplasmic abscission of the ectopic polar body induced by sperm chromatin-spindle. (A–C) Different confocal sections of an egg showing ectopic polar body at 7 o'clock position (arrows) and PbII at the 11 o'clock position. Note the complete separation of cytoplasm membrane between ectopic polar body (C arrow) and the fertilized egg. The sperm chromatin-injected egg was activated by IVF and the extra DAPI staining spots are surface bound sperm. (1.10 MB DOC) [file pone.0007171.s001.doc]

**
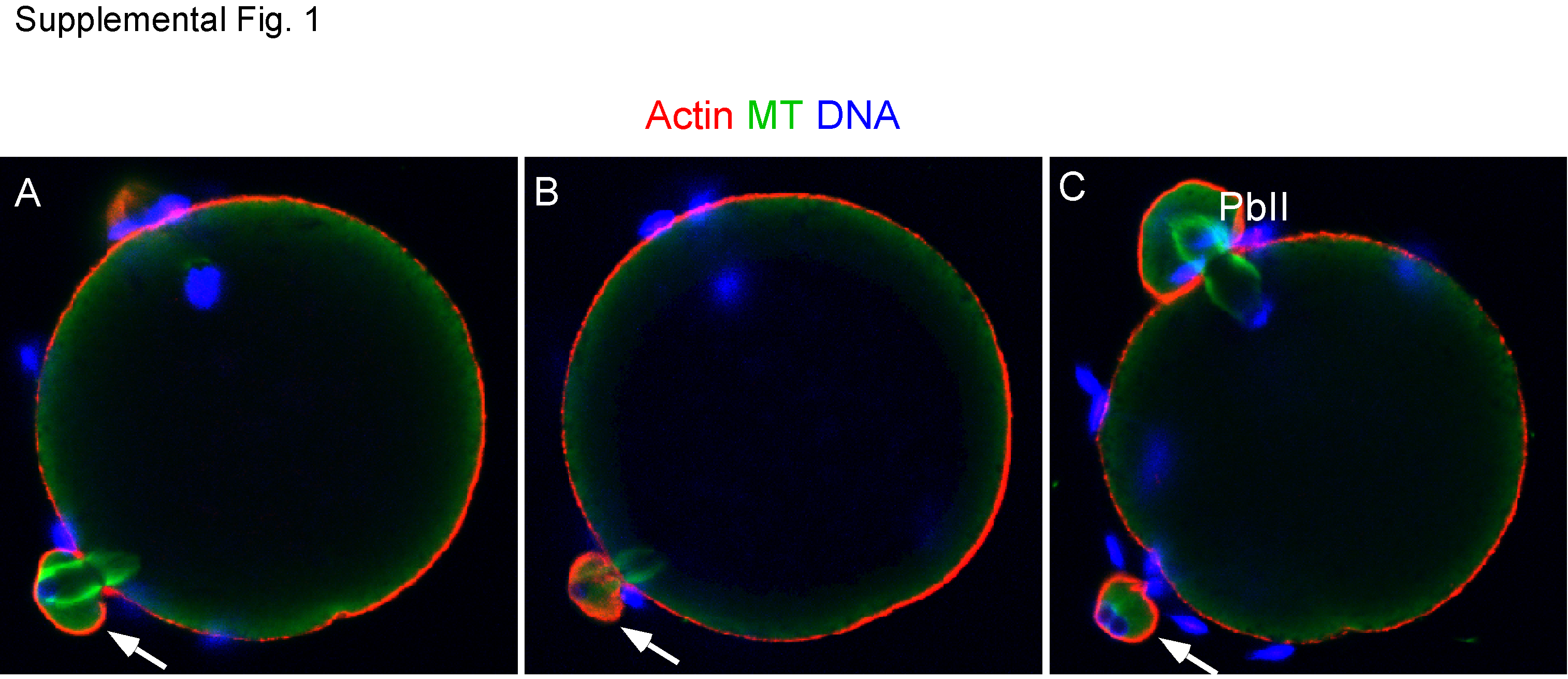
**

Figure S1. Confocal images showing the complete cytoplasmic abscission of the ectopic polar body induced by sperm chromatin-spindle. (**A-C**) Different confocal sections of an egg showing ectopic polar body at 7 o’clock position (arrows) and PbII at the 11 o’clock position. Note the complete separation of cytoplasm membrane between ectopic polar body (C arrow) and the fertilized egg. The sperm chromatin-injected egg was activated by IVF and the extra DAPI staining spots are surface bound sperm.
